# Promoting RNA editing by ADAR attraction

**DOI:** 10.1186/s13059-017-1343-7

**Published:** 2017-10-23

**Authors:** Miri Danan-Gotthold, Erez Y. Levanon

**Affiliations:** The Mina and Everard Goodman Faculty of Life Sciences, Bar-Ilan University, Ramat-Gan, 52900 Israel

## Abstract

Concentration is important and not only while driving; a new study indicates how an adjacent genomic element helps to increase the efficiency of a specific adenosine to inosine RNA editing reaction, by providing a means to increase the local concentration of the RNA editing enzyme ADAR.

## Introduction

RNA editing by base deamination is an endogenous process of site-directed mutagenesis employed by organisms in all metazoa to modify genetic information as it passes through RNA. Thus, RNA deaminases such as APOBEC1 and the adenosine deaminase that acts on RNA (ADAR) family of enzymes can potentially become tools for manipulating genetic information by modifying the RNA sequence. This has the major advantage over other systems such as CRISPR/Cas9 in that the genetic information of the cell remains untouched and no introduction of a foreign protein (CAS9) into cells is required. However, the current limited knowledge about the factors that determine editing target selection hinders the ability to utilize them efficiently. The manuscript by Daniel et al. [1] reveals a novel key component needed for efficient RNA editing [2, 3].

## RNA editing targets

In mammals, ADAR1 and ADAR2 are two functional adenosine deaminases that act on RNA [4]. These enzymes deaminate adenosine residues to inosines in a reaction known as adenosine to inosine (A-to-I) RNA editing, which is the most prevalent transcriptional modification in human cells [5]. Inosine is recognized by most biological machineries as guanosine (G), and hence editing can alter the protein-coding outcome, generating proteomic and phenotypic diversity. Most A-to-I editing occurs in double-stranded RNA (dsRNA) sequences and mainly in untranslated regions (UTRs) and introns, where large duplexes may be formed by nearby reversely oriented mobile elements. Although mostly promiscuous in the context of long dsRNAs, A-to-I editing is also highly specific within several evolutionary conserved RNA structures, resulting in the editing of a single or limited number of adenosine residues within targets such as glutamate receptor subunit GluA2 and potassium channel transcript Kv1.1. Such sites are found mainly in coding regions and the editing frequently changes the protein sequence (recoding) [6]. The extent of RNA editing in these sites does not always directly correlate with the ADAR expression levels. Deciphering what determines the specificity and efficiency of editing in these sites has far-reaching implications but currently this process is poorly understood [7].

## Editing is regulated by distant structural elements

**Fig. 1:**
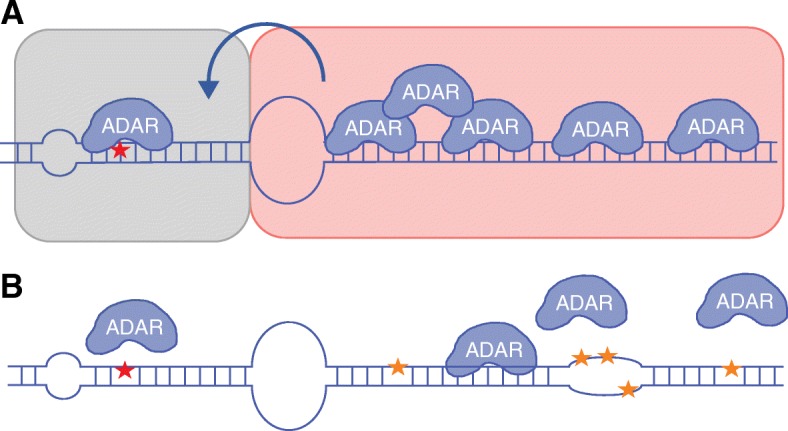
Editing inducer elements (EIEs) regulate specific adenosine to inosine (A-to-I) editing. **a** Structural elements that contribute to selective and efficient editing around the edited adenosine (*red star*): short imperfect stem (*gray background*), internal loop and EIE identified in the study by Daniel et al. [1] (*red background*). The suggested model proposes ADAR (adenosine deaminase that acts on RNA) recruitment to the EIE, thereby increasing the local concentration in the vicinity of the edited RNA molecule. This improves the conditions for catalysis and promotes efficient editing at the specific site. **b** A high local concentration of ADARs may result in hyperediting of the long double-stranded RNA (*orange stars*). Because the hyperedited double-stranded RNA is less favorable for ADAR binding, the local concentration of ADARs decreases

Originally, only the primary sequence and the structure adjacent to a specific edited site were thought to affect editing specificity and efficiency, such as the sequence preference of ADAR and an imperfect helical structure neighboring the edited adenosine [8]. However, in 2012, a structure at a distance from the edited adenosine stem was shown to increase the editing efficiency and specificity of a highly edited site in the gene Gabra-3 [9]. Daniel et al. extend their initial observation and describe editing inducer elements (EIEs) as a general mechanism contributing to efficient editing at specific sites. These elements include stem structures, which are separated from the main target stem by a long internal loop (Fig. 1a). EIEs for several efficiently edited adenosine residues were identified in the study and were shown to induce editing independent of their sequence and location upstream or downstream to the edited adenosine. This suggests that the increased editing efficiency probably results from recruiting ADAR enzymes to the RNA molecule. Moreover, the large loop separating the EIE from the edited site stem was shown to contribute to the site selectivity by limiting the editing of adenosine residues adjacent to the specific site.

## Possible mechanism of regulating editing levels by EIEs

The unique mechanism suggested by Daniel et al. [1] is reminiscent of an aircraft carrier which serves as a convenient landing field for planes. In this case, EIEs on the RNA molecule provide a highly efficient binding site, which recruits multiple ADARs to a location in proximity to the specific target. Thus, the study indicates a new important role for long dsRNAs and dsRNA binding domains (dsRBDs) of ADAR in increasing the local concentration of enzyme in the vicinity of the site targeted for editing. This results in highly efficient editing at the selected site.

Since the long dsRNA is also promiscuously edited by ADAR enzymes, a reasonable speculation may be that these elements also have a role in releasing the ADARs from the RNA molecule. In this way, a molecule that has attracted a very high concentration of enzyme will be eventually hyperedited at the long stem, lowering the ability to recruit further ADARs (Fig. 1b).

Long dsRNAs are largely composed of reversely oriented non-coding retrotransposons, which constitute approximately half of the mammalian genome. The effect of these elements in the cell is mostly unclear and they are generally considered non-functional. Daniel et al. provide a new noteworthy regulatory meaning for some of these mobile elements by showing they induce RNA editing activity. This may be also valid for additional dsRNA-binding proteins, potentially extending the role of these elements.

## Conclusions

A-to-I editing has been studied extensively and shown to play a role in developmental processes and disease. Aberrant editing has been associated with autoimmune disorders, cancer, and neurological disorders [10]. Here, Daniel et al. [1] add another significant piece of knowledge to the mechanism of specific substrate recognition by ADAR enzymes. A better understanding of this mechanism can facilitate the important task of identifying novel editing sites and also aid in understanding the cause of aberrant editing in diseases. Finally, an increased understanding of this mechanism opens the way for the development of RNA-editing tools for research and disease treatment, with the potential for better safety compared with the current DNA-based genomic-editing tools.

## Abbreviations

ADAR: Adenosine deaminase that acts on RNA
A-to-I: Adenosine to inosine
dsRNA: Double-stranded RNA
EIE: Editing inducer element
